# Dietary Natural *N*-Acetyl-d-Glucosamine Prevents Bone Loss in Ovariectomized Rat Model of Postmenopausal Osteoporosis

**DOI:** 10.3390/molecules23092302

**Published:** 2018-09-09

**Authors:** Zhiwen Jiang, Zhe Li, Wei Zhang, Yan Yang, Baoqin Han, Wanshun Liu, Yanfei Peng

**Affiliations:** 1 Laboratory of Biochemistry and Biomedical Materials, College of Marine Life Sciences, Ocean University of China, Qingdao 266003, China; jiangzhiwen@ouc.edu.cn (Z.J.); zhangwei0874@sina.cn (W.Z.); yany@ouc.edu.cn (Y.Y.); baoqinh@ouc.edu.cn (B.H.); WanshunLiu@hotmail.com (W.L.); 2 Laboratory for Marine Drugs and Bioproducts of Qingdao National Laboratory for Marine Science and Technology, Qingdao 266235, China; 3 Qingdao Biotemed Biomaterial Co., Ltd., Qingdao 266101, China; 15192608167@163.com

**Keywords:** *N*-acetyl-d-glucosamine, postmenopausal osteoporosis, ovariectomized, osteoblasts

## Abstract

Postmenopausal osteoporosis has seriously affected the life quality of elderly women. A natural polymer, chitin, obtained from shells of crab and shrimp, has been widely used in the biomedical field owing to its nontoxicity, biocompatibility, and biodegradability. In this study, natural *N*-acetyl-d-glucosamine (NAG) was prepared from liquefied chitin. The protective activities of NAG in postmenopausal osteoporosis were evaluated on Sprague Dawley rats and osteoblast-based models. Results showed that oral administration of NAG boosted trabecular bone volume and trabecular numbers. Additionally, the calcium content in the femur and tibia increased, and femoral biomechanical properties improved. Furthermore, NAG supplementation significantly lowered alkaline phosphatase levels and increased calcium content in the serum of ovariectomized rats. In vitro studies showed that NAG markedly promoted cell proliferation and stimulated osteoblast differentiation of mouse calvaria origin MC3T3-E1 cells with increased alkaline phosphatase activity in a concentration-dependent manner. Moreover, NAG effectively protected osteoblasts from oxidative damage induced by hydrogen peroxide. In conclusion, our data provide an additional foundation for dietary supplementation of NAG, which could protect and reverse osteopenia in postmenopausal women.

## 1. Introduction

Osteoporosis (OP), defined as a systemic skeletal disorder characterized by compromised bone strength, decreased bone mass, microarchitectural deterioration, and increased fragility, predisposes the patient to an increased risk of bone fracture [1,2]. Elder women easily suffer from postmenopausal osteoporosis (PMOP), caused by aging, continuous calcium loss, and estrogen deficiency [3,4]. PMOP, a multifactor disease relevant to nutrition of racial genetic factors and mode of motion, is a major female health problem that increases morbidity, mortality, and healthcare system costs [5]. Various medicines have been developed for the prevention and treatment of PMOP. Bisphosphonates are the first choice of drug therapy as they can inhibit the re-absorption of bone by promoting the apoptosis of osteoclasts [6,7]. However, bisphosphonates can increase the accumulation of bone microdamage, and have been found to cause atypical stress fractures of the midshaft or subtrochanteric femur [8,9,10]. Hormone replacement therapy is another choice for PMOP [11]; however, hormone replacement has side effects that can cause many complications in the human body. Currently, anti-PMOP compounds composed of natural non-toxic material with bone inductive activity are being investigated.

Chitin, the most abundant natural polymer on the earth except for cellulose, obtained from shells of crab and shrimp, has been widely used in the biomedical field owing to its nontoxicity, biocompatibility, and biodegradability [12,13]. Chitin is a water insoluble polymer, which is the major limiting factor for its utilization in living systems. Our study focused on *N*-acetyl-d-glucosamine (NAG), the monosaccharide of chitin, that is acetylated on the amino group [14]. Due to the small molecular weight, water solubility, good biological activities, absorption, and utilization in the body, NAG overcomes the technical problems associated with chitin [15]. In addition, NAG is the basic unit of many important polysaccharides in biological cells, such as hyaluronic acid and keratin sulfate. NAG has been reported to facilitate hyaluronic acid synthesis and improve wound healing. Furthermore, NAG possess anti-oxidant and immune-modulating effects and can regulate liver function and treat immuno-hepatic injury in clinical settings [16,17]. There has been an increasing demand for NAG being used in natural food additives.

Although the effects of NAG on the cure of bone fracture has been reported [18,19], studies focusing on bone-associated diseases are lacking. The production of NAG by chemical methods is economic, whereas the product has not yet received approval as a natural material due to its chemical modification [20,21]. Furthermore, the large quantities of chemical waste resulting from chemical processes are not environment-friendly [22]. In the present study, enzymatic hydrolysis was adopted to produce natural NAG using chitin from snow-crab shell as the substrate. Moreover, the role of NAG in treatment of PMOP was evaluated in Sprague Dawley rats and osteoblast-based models. Our data provide a basis for developing a more effective and non-toxic natural dietary supplement against PMOP. 

## 2. Results 

### 2.1. Identification and Properties Analysis of NAG 

**Figure 1:**
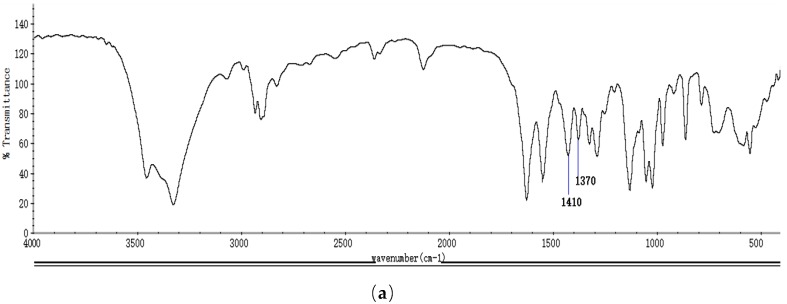

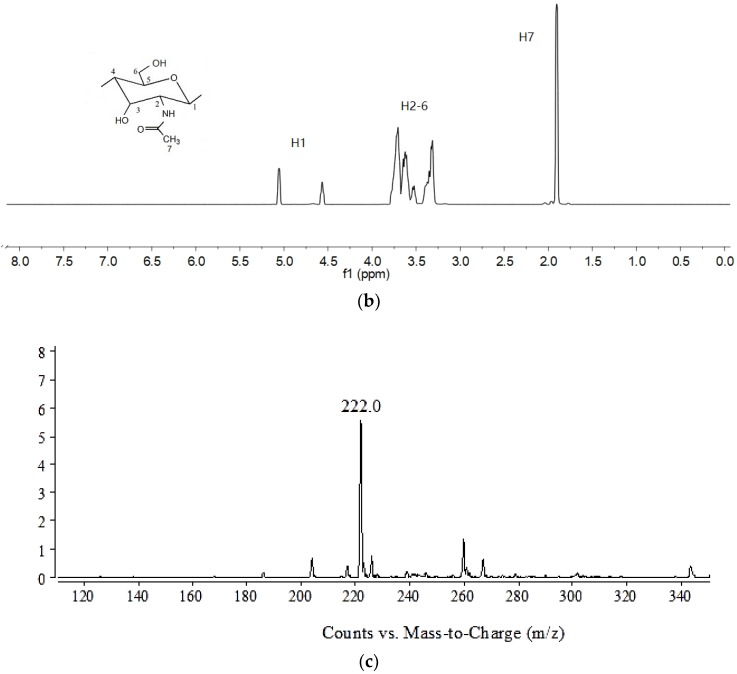
(**a**) Infrared (IR) spectra, (**b**) proton nuclear magnetic resonance (^1^H-NMR) spectra, and (**c**) mass spectrometry (MS) of *N*-acetyl-d-glucosamine (NAG).

As shown in the Fourier transform infrared (FTIR) spectroscopy spectrum of NAG (Figure 1a), absorption peaks at 1650 cm^−1^, 1550 cm^−1^, and 1310 cm^−1^ were due to the asymmetric and symmetrical stretching vibrations of amide, and the absorption peaks at 1410 cm^−1^ and 1370 cm^−1^ were the presence of the acetyl group, which confirmed the acetylation of glucosamine. As shown in proton nuclear magnetic resonance (^1^H NMR) spectra of NAG (Figure 1b), the signal of H7 was assigned to the *N*-acetyl group of NAGS, and signals at δ5.2 ppm and δ4.7 ppm were attributed to H1 of α- and β-anomers of NAG, respectively. The sole monosaccharide was identified in the mass spectrum (MS) of NAG (Figure 1c). The prepared NAG was determined to have a molecular weight of 221 Da with 10% water content, 0.2% ash content, and 98.8% purity.

### 2.2. Dietary NAG Ameliorated Body and Uterine Weight of Ovariectomized Rats 

**Figure 2:**
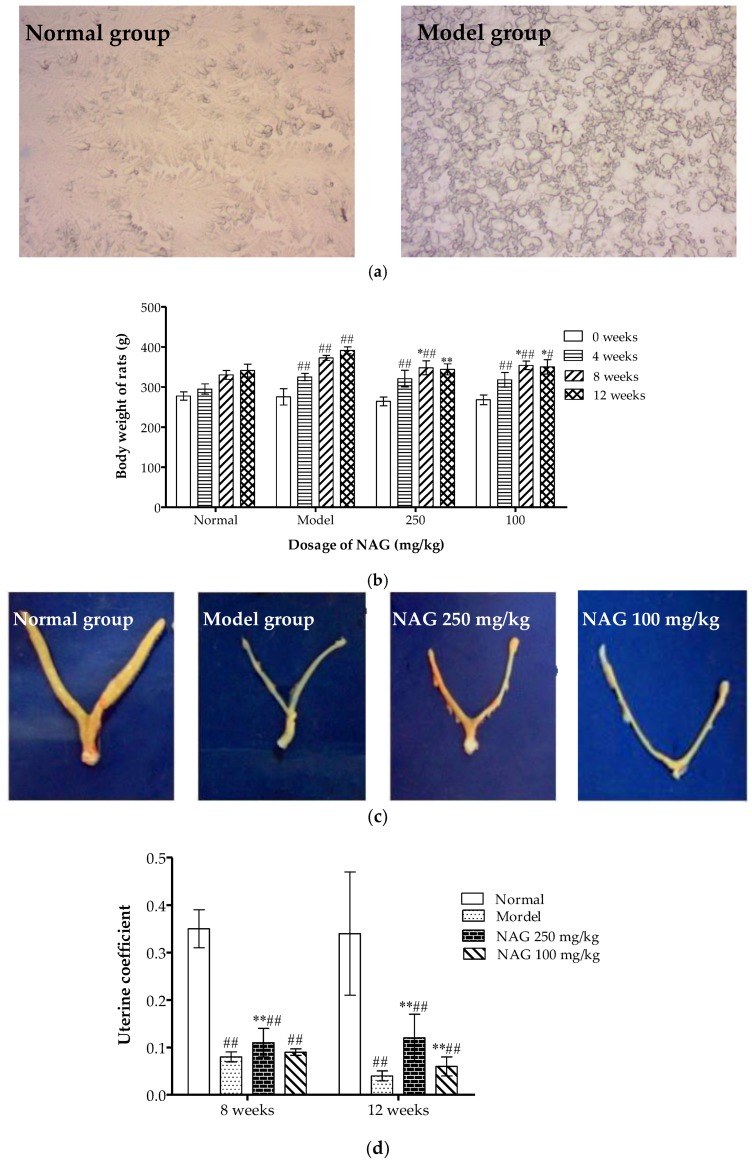
Effects of NAG on body and uterine weight of ovariectomized rats. (**a**) Morphological changes of vaginal smears two weeks after Sprague-Dawley (SD) rats were ovariectomized (original magnification, 200×). (**b**) Body weight of all rats measured during the 12-week treatment course. (**c**) Photos of uterine horns of all rats after 12 weeks’ treatment. (**d**) Uterine coefficient of all rats after 8 and 12 weeks’ treatment. Data represent mean ± SD, *n* = 6; ^#^ *p* < 0.05 and ^##^ *p* < 0.01: significant difference compared with normal group; * *p* < 0.05 and ** *p* < 0.01: significant difference compared with model group.

Vaginal smears were diagnosed two weeks after transplantation to confirm the removal of ovaries. As shown in Figure 2a, many compressed and translucent epidermal cells were observed in normal rat vaginal smears. In the model group, obvious cell fragmentation was observed with no significant estrous cycle, which confirmed the ovariectomy of Sprague-Dawley (SD) rats. The body weight of all rats during the 12-week treatment course is illustrated in Figure 2b. Significantly increased body weight was found in all ovariectomized rats at four weeks compared with normal rats (*p* < 0.01). Body weight significantly decreased in NAG-treated groups after eight weeks of treatment compared with the model group (*p* < 0.05). The body weight of animals treated with high doses of NAG reached those of the normal group at 12 weeks (*p* > 0.05). Ovariectomy was validated by atrophy of the uterine horns for all ovariectomized rats, and a significantly restored uterus was found in the NAG group (250 mg/kg) as shown in Figure 2c,d. 

### 2.3. NAG Consumption Changed Alkaline Phosphatase and Ca Contents in Serum of Ovariectomized Rats

**Figure 3:**
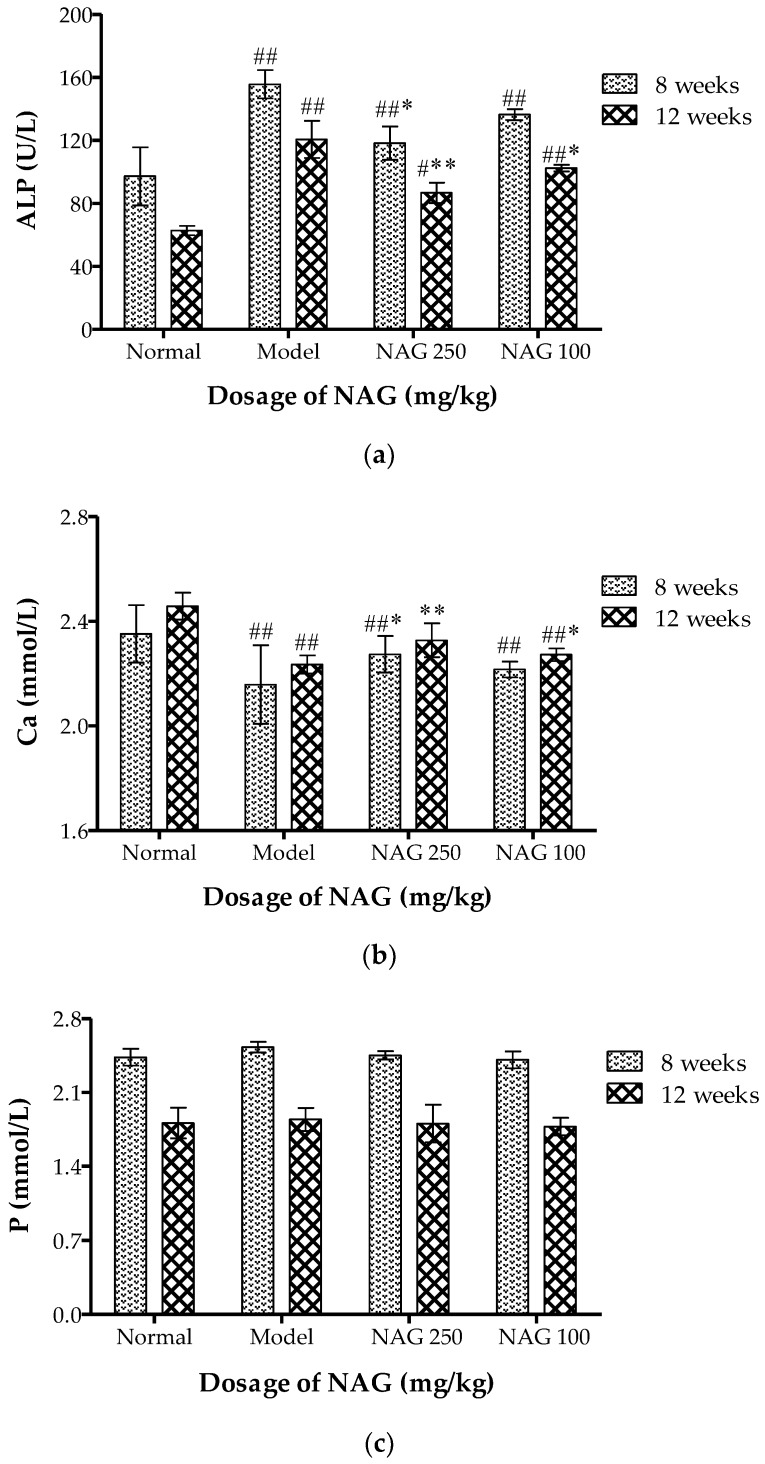
Effects of NAG on serum biochemical parameters of ovariectomized rats. Data represent mean ± SD; *n* = 6; ^#^ *p* < 0.05 and ^##^ *p* < 0.01: significant difference compared with normal group; * *p* < 0.05 and ** *p* < 0.01: significant difference compared with model group.

Compared with the normal group, a significant increase in serum alkaline phosphatase (ALP) occurred in the model group (*p* < 0.01). NAG significantly decreased serum ALP levels (Figure 3a) in the ovariectomy-induced bone loss rats in a dose- and time-dependent manner (*p* < 0.01). A highly significant increase in serum calcium was observed in all experimental groups compared with model group at 12 weeks. Serum calcium returned to normal in the NAG (250 mg/kg) treated group (Figure 3b). As shown in Figure 3c, there was no significant difference in serum levels of phosphorus in all groups.

### 2.4. NAG Administration Improved Bone Mechanical Properties of Ovariectomized Rats 

As shown in Table 1, the maximum load and fracture deflection significantly decreased in ovariectomized rats. The maximum load (N) in NAG groups was 71.97% and 58.18%, which were significantly higher than that of the model group. Our results also showed higher relative fracture deflection in the NAG (250 mg/kg) treated group compared with the model group (*p* < 0.01). Compared with the model group, there was a significant increase (*p* < 0.05) in maximum load and fracture deflection in high NAG doses group on the eighth week. NAG increased mechanical strength in a dose-dependent manner at 8 and 12 weeks.

### 2.5. Elevation of Bone Mineral Calcium Content in Femur and Tibia by NAG 

As shown in Table 2, femoral dry weight, ash weight, and calcium content were measured to further illustrate the effects of NAG treatment on mineral content in rats. The femoral dry weight, ash weight, and inorganic content in the model group were significantly reduced compared with the normal group (*p* < 0.01). Bone dry weight and ash weight after oral administration of NAG for 12 weeks were higher than that in model group in a dose-dependent manner. The highest inorganic content in the NAG groups was 65.93% (*p* < 0.01). Bone calcium contents in the NAG groups were all significantly higher (*p* < 0.01) than those of the model group. However, the calcium content in the femurs was higher than in the tibias.

### 2.6. Antiosteoporotic Activity of NAG in Tibia Bones of Ovariectomized Rats

**Figure 4:**
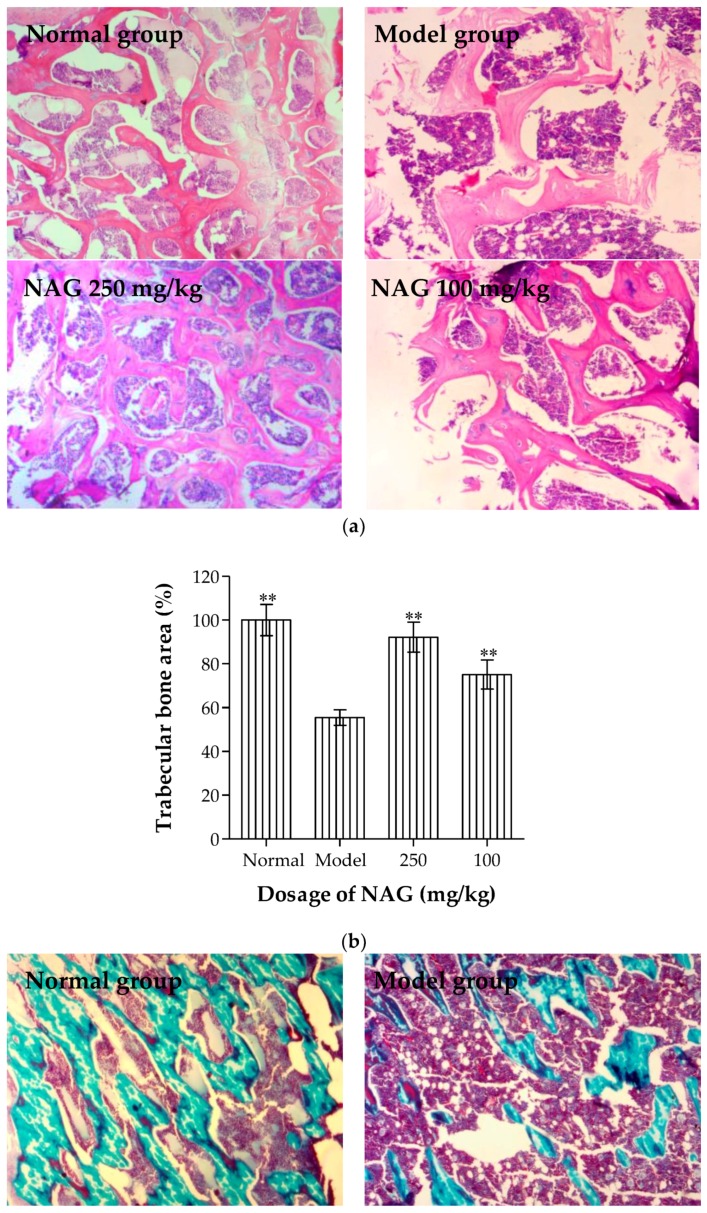

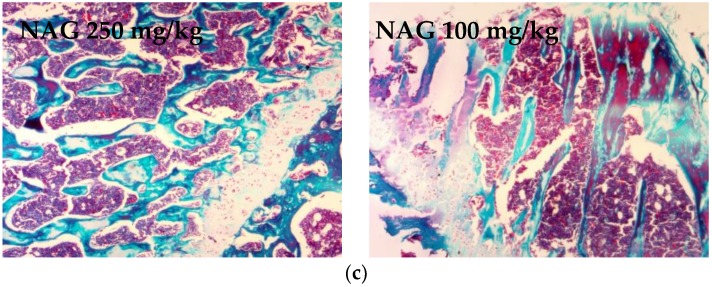
(**a**) Histopathological changes in transverse tibia tissue obtained via hematoxylin and eosin (H&E) staining, (**b**) quantified trabecular bone area, and (**c**) Masson trichrome staining. Osteoblast, osteoclast, and empty lacunae are marked with black, red, and white arrows in H & E-staining (original magnification, 200×), respectively. Data represent mean ± SD; *n* = 6, ** *p* < 0.01: significant difference compared with model group.

Histological sections of the tibia in Figure 4 show a decrease in trabecular volume as well as disconnection of trabeculae among the ovariectomized rats. Lacunae and osteoclasts were localized in trabecular bone, and osteoblasts were adjacent to the neoformed bone trabecular. Incomplete trabecular structure, a large amount of empty bone lacunae, fibrotic marrow, shrunk nuclei, and dissolved osteocytes were observed under an electron microscope in the model group. The increased osteoblasts and trabecular bone area, and decreased osteoclasts and lacunae were observed by hematoxylin and eosin (H & E) staining in NAG groups when compared with the model group (Figure 4a,b). Trichrome staining results (Figure 4c) showed that the bone trabecular of the normal group grew normally in accordance with the close-packed rule, which were thick and plump with prominent osteoblasts and tissue structural integrity. Conversely, bone trabeculars in the model group had a sparse and inconsecutive array compared with the normal group. NAG treatment had a positive effect on the connectedness and overall number of bone trabeculae.

### 2.7. Elevation of Osteoblasts Proliferation and Differentiation by NAG

**Figure 5:**
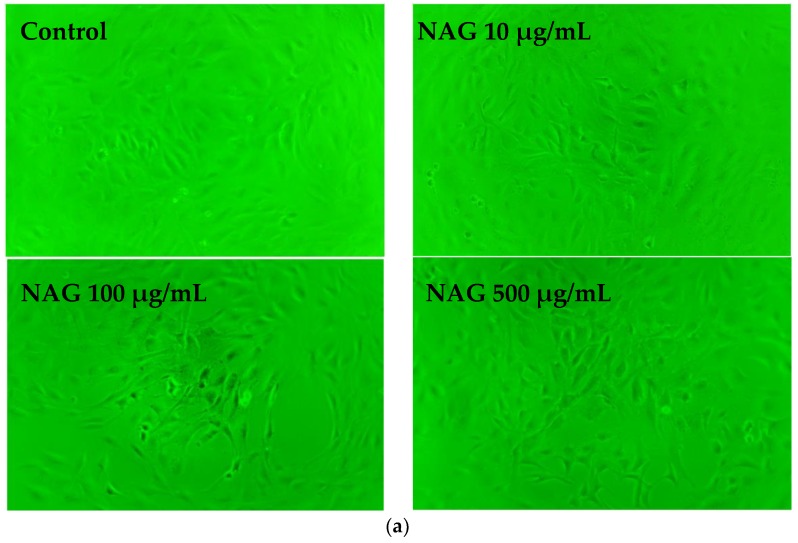

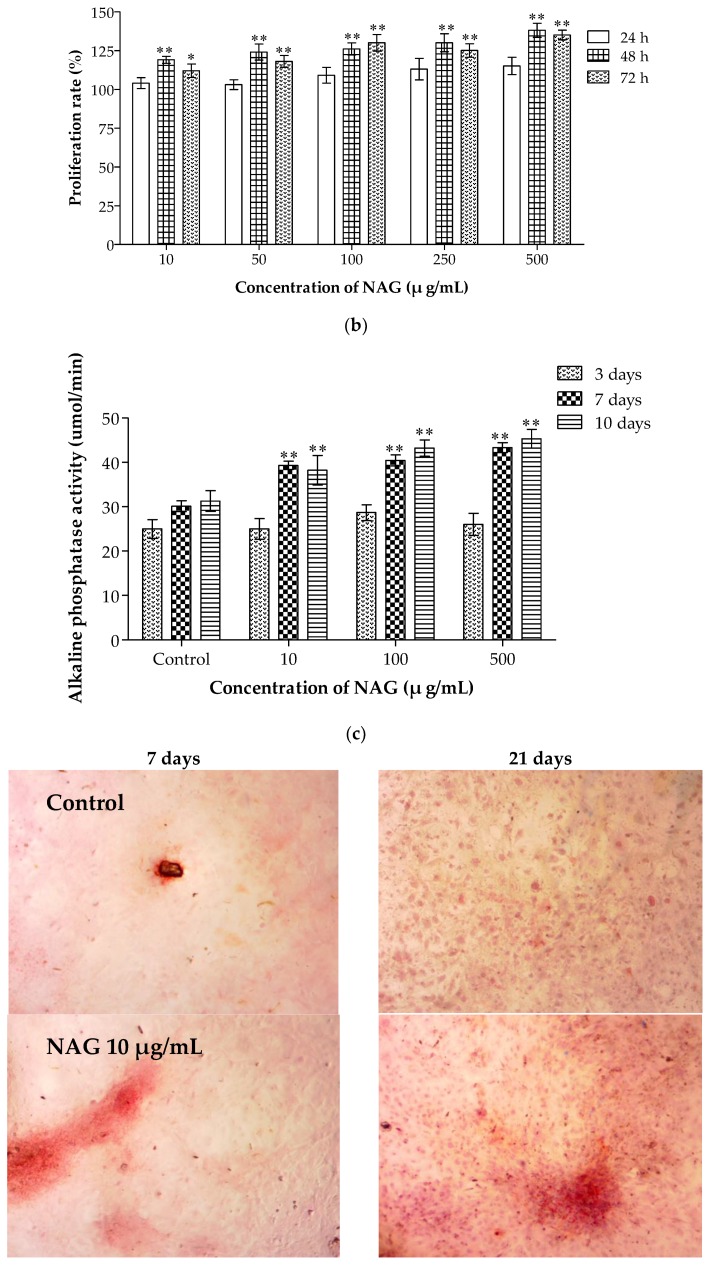

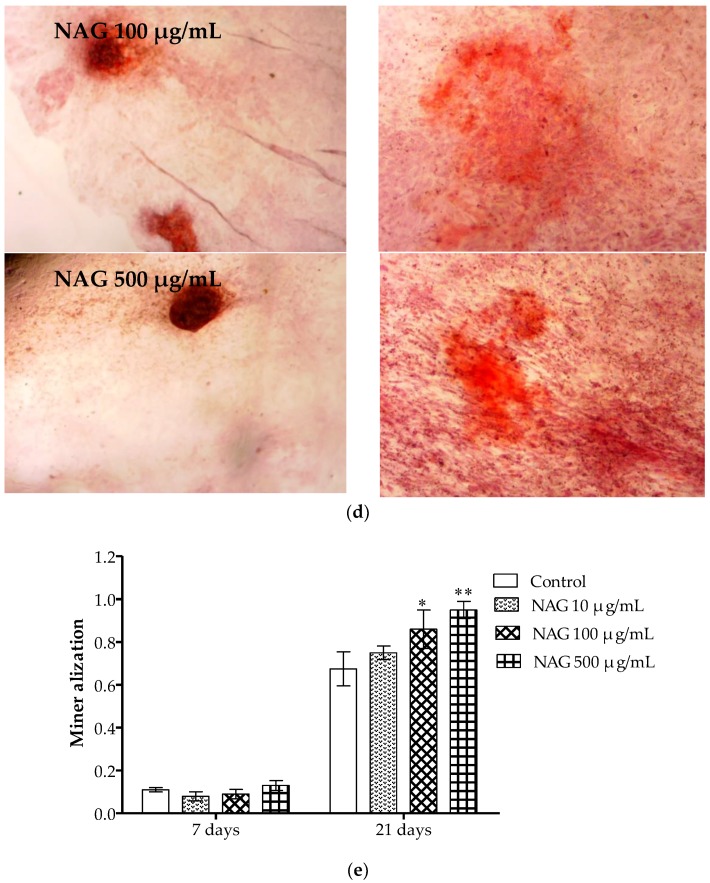
Effects of NAG treatment on proliferation and differentiation of MC3T3-E1 cells. (**a**) Morphological changes of MC3T3-E1 cells were examined under a light microscope at 48 h (original magnification, 200×). (**b**) Proliferation rate measured by 3-(4,5-dimethylthiazol-2-yl)-2,5-diphenyl tetrazolium bromide (MTT) test at 24, 48, and 72 h. (**c**) alkaline phosphatase (ALP) activities in MC3T3-E1 cells. (**d**) Alizarin Red S staining of MC3T3-E1 cells. (**e**) Quantitative results of cell mineralization. Data represent mean ± SD; *n* = 6; * *p* < 0.05, ** *p* < 0.01: significant difference compared with control group.

The differences in morphology and cell proliferation between control group and NAG treated groups were compared. As shown in Figure 5a, cells in each group spread well and the cytoplasm was rich after NAG incubation for 48 h. 3-(4,5-dimethylthiazol-2-yl)-2,5-diphenyl tetrazolium bromide (MTT) results (Figure 5b) showed that proliferation of MC3T3-E1 osteoblasts increased in a concentration- and time-dependent manner after NAG incubation (*p* < 0.05). After 48 h, cell proliferation increased by 30% with treatment of NAG (500 μg/mL) when compared with the control group (*p* < 0.01). Our results showed that there was no significant difference in ALP activity between control group and NAG-treated groups at three days. ALP activities significantly increased in concentration- and time-dependent manners at 7 and 10 days when compared with the control group (*p* < 0.01) (Figure 5c). The Alizarin Red S staining results showed that more mineralized nodules were observed in MC3T3-E1 cells treated with NAG at days 7 and 21 than in the control group (Figure 5d). The numbers of mineralized nodules also showed a significant increase in a concentration- and time-dependent manner (Figure 5e).

### 2.8. NAG Protects MC3T3-E1 Cells against H_2_O_2_ Oxidative Damage

**Figure 6:**
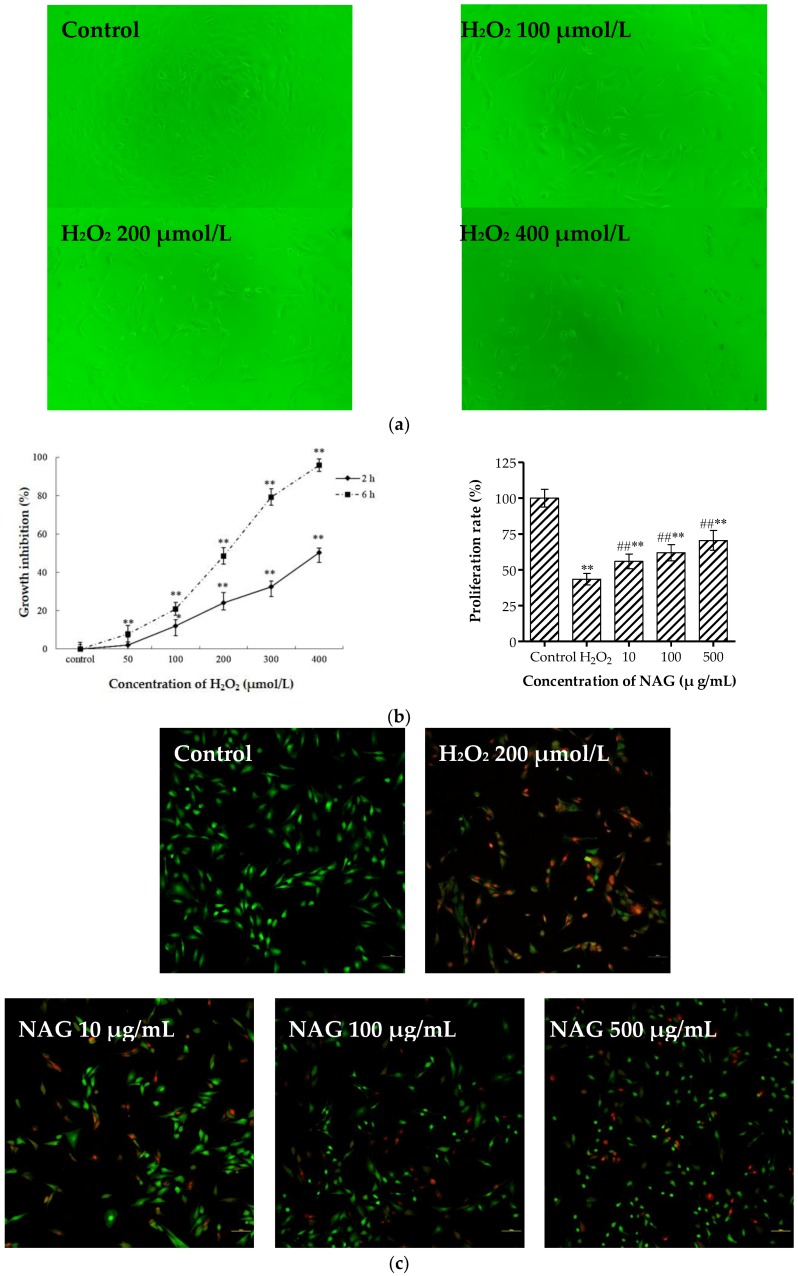
Protective functions of NAG on MC3T3-E1 cells from hydrogen peroxide (H_2_O_2_). (**a**) Morphological changes of injured MC3T3-E1 cells were examined under a light microscope at six hours (original magnification, 100 ×). (**b**) Growth inhibition of H_2_O_2_ and repairing effect of NAG on injured MC3T3-E1 cells measured by MTT assay. (**c**) Calcein/propidium iodide (PI) stain of injured MC3T3-E1 cells were examined under a fluorescence microscope (original magnification, 200×). Data represent mean ± SD, *n* = 6; * *p* < 0.05, ** *p* < 0.01: significant difference compared with control group; ^##^ *p* < 0.01: significant difference compared with H_2_O_2_-injuried group.

Incubation with hydrogen peroxide (H_2_O_2_) resulted in changes in the shapes and fine structures of osteoblasts observed by inverted microscope (Figure 6a). MTT results (Figure 6b) showed that H_2_O_2_ (50, 100, 200, 300, and 400 μmol/L) significantly reduced cell viability in a concentration-dependent manner at two and six hours, and cell mortality was controlled at 50% by H_2_O_2_ (200 μmol/L). Therefore, H_2_O_2_ at a concentration 200 μmol/L was selected as the model concentration for six hours, which was consistent with previous studies [23,24,25]. As shown in Figure 6b,c pretreatment with different concentrations of NAG for 24 h significantly attenuated H_2_O_2_-mediated cell death. Propidium iodide (PI)/calcein staining also showed that the cell survival rate significantly increased compared to the H_2_O_2_ group.

## 3. Discussion 

Postmenopausal osteoporosis, a common age-related bone disease, is a major public health threat to elderly women. Therefore, development of natural non-toxic compounds for prevention is highly desirable. NAG, a monosaccharide of chitin generated by hydrolysis, is categorized as a hexosamine, which has been the focus, along with d-glucosamine, for the improvement of osteoarthritis [14]. In the present study, natural NAG was prepared from liquefied chitin with hydrolases and the chemical structure was elucidated by FTIR and ^1^H-NMR. Moreover, NAG was investigated as a preventative compound for PMOP in SD rats and osteoblast-based models.

The pathogenesis of postmenopausal osteoporosis is complicated. Due to the decline of bone biomechanical properties induced by low levels of estrogen, postmenopausal women present with an increased risk of osteoporosis. Therefore, the establishment of an animal model of postmenopausal osteoporosis is often based on spontaneous or induced ovarian dysfunction. The reproductive organ and body weight of three-month-old female rats have become more mature, and the bone metabolism is in a relatively balanced state. A rat osteoporosis model caused by ovariectomy has been established to replicate clinical features of osteoporosis in postmenopausal women. 

Ovariectomized animals and many postmenopausal women show increased body weight and white adipose tissue, which is called metabolic obesity [26]. Dietary NAG supplementation was effective in ameliorating ovariectomy-induced obesity, which makes it a promising treatment for postmenopausal obesity. Estrogen secretion in SD rats decreases after ovariectomy, which produces endocrine dyscrasia that causes a series of bone metabolism disturbances. Firstly, the balance between bone formation and bone resorption is broken, then loss of bone mass urges the animal to gain weight to remedy the loss. Consequently, weight increases rapidly in a process called metabolic obesity. Our results showed that NAG treatment could prevent ovariectomy-induced weight increase and uterine atrophy.

ALP is a common marker for evaluating bone formation and bone transformation as ALP is released into the blood when estrogen secretion is disordered [27]. Hence, the increase in serum ALP enzyme activity can reflect the activity of the osteocyte metabolic process. The content of calcium and phosphorus in the blood can reflect the bone’s physical structure to some degree. In our experiment, the ALP activity in the NAG-treated group at week 12 decreased by 40% compared to week eight, which roughly returned to the level of the normal control group. Contents of calcium and phosphorus were also maintained at a certain level. Our data imply that NAG positively regulates changes in bone metabolism caused by decreased estrogen level in ovariectomized rats. 

The inorganic constituents in bone are responsible for the solid tissue nature of bone, whereas the organic constituents imbue certain suppleness. When a bone suffers external force, its inner structure and external form alter accordingly. The alteration is reflected in the changes in structural mechanics and the material mechanics of bone. Three-point bending is the best index for testing bone biomechanics [28]. Thus, the risk of fragility fracture could be evaluated. Our results showed that the maximum load and fracture deflection declined apparently after the gonadectomy indicating that the strength and toughness of bones were destroyed. The bone load and deflection were significantly improved after oral NAG administration for 12 weeks, suggesting that the bone fracture was avoided after long-term treatment with NAG.

The existence of inorganic ingredients can make bone hard and firm. Both decreased bone mineral density and inorganic substances can result in the decrease in bone strength and properties. With the decrease in estrogen and increasing activity of osteoclasts, precious inorganic calcium in bone tissue decreases sharply due to the dissolution of bones. However, our present study found that this decrease was quite different between the femur and tibia. The calcium decreased by 32.8% in the femur, whereas the calcium was decreased by 42.38% in the tibia. This may be due to the differences in their physiological structures. Besides, we found that NAG treatment significantly increased the content of inorganic substances in the femur. The inorganic content in normal bone tissue is about 65%, whereas that in the control group was obviously low. We concluded that NAG treatment can effectively increase the mineral content due to its small molecular weight, ease of permeating cell membranes, and promotion of bone growth through the combination of ALP and inorganic salts (in the form of hydroxyapatite crystals). Previous studies found that the mixture of hydroxyapatite and chitosan was easily injected into bone marrow and promoted bone growth [29]. 

The effect of NAG on the histopathological changes of tibia bones was examined by H & E and trichrome staining, which characterize bone impairment and pathogenesis. Treatment with NAG preserved the thickness and density of bone trabecular when compared with the model group. The results showed that oral NAG administration was effective in ovariectomized-induced bone loss by promoting osteoblasts activity and increasing the compactness of trabecular bone.

In the previous part of this paper, the effects of NAG treatment on postmenopausal osteoporosis were observed in adult ovariectomized rats. Postmenopausal osteoporosis results from a disruption of the fine balance between bone formation and resorption [30]. The MC3T3-E1 cell line, derived from newborn murine calvariae, retains osteoblast-like characteristics after repeated passages [31]. The following study will focus on the influence of NAG on the in vitro proliferation, differentiation, and mineralization of osteoblasts. ALP activity is an early marker of osteogenic differentiation [32,33,34]. Extracellular matrix mineralization is also a major part of late bone formation [35]. NAG treatment showed significant stimulatory effects on proliferation, ALP activity, and mineralization of MC3T3-E1 cells, which highlights its potential in bone formation promotion. Reactive oxygen species (ROS) are chemically reactive molecules generated from oxygen [36,37]. During the process of bone remodeling, H_2_O_2_ produced by osteoclasts could damage and even cause death to osteoblasts. This would result in bone re-absorption other than bone formation, and finally causing PMOP. In our study, NAG exhibited excellent antioxidative activities significantly protect MC3T3-E1 cells against H_2_O_2_ in a concentration-dependent manner. 

## 4. Materials and Methods 

### 4.1. Materials and Reagents 

ALP assay kit, Bradford protein assay kit, and Triton X-100 were purchased from Beyotime Bioengineering Institute (Shanghai, China). Masson Stain Kit, calcium (Ca) assay kit, and phosphate assay kit were obtained from Jiancheng Bioengineering Institute (Nanjing, China). Calcein, propidium iodide, sodium pentobarbital, and 3-(4,5-dimethylthiazol-2-yl)-2,5-diphenyl tetrazolium bromide (MTT) were obtained from Sigma-Aldrich (St. Louis, MO, USA). Modified essential medium Alpha Modification (MEM-Alpha) and fetal bovine serum (FBS) were purchased from Gibco^®^, Life Technologies (Carlsbad, CA, USA). All chemicals were of analytical grade and received from Sinopharm Chemical Reagent Co., Ltd. (Shanghai, China), unless otherwise indicated.

### 4.2. Preparation and Physico-Chemical Characteristics of NAG 

NAG was extracted from fermentation broth by reduced pressure concentration, decolorization with activated charcoal, and crystallization, and refined with ethyl alcohol in our laboratory. Molecular weight was determined by high performance liquid chromatography (HPLC). NAG structure was characterized by FTIR, ^1^H-NMR, and MS. The IR spectrum of NAG (~0.5 mg) was acquired with a NEXUE 470 instrument (Nicolet Co. Madison, WI, USA) as KBr pellets at room temperature. ^1^H-NMR spectra of NAG were recorded on a DPX 300 spectrometer (Bruker, Rheinstetten, Germany) using acetone as internal standard and D_2_O as solvent at 25 °C. The MS of NAG was detected with an Agilent 6460 Triple Quad LC/MS system (Agilent Technologies Inc. Santa Clara, CA, USA). 

### 4.3. Animals and Feeding 

Twelve-week-old female SD specific-pathogen-free (SPF) rats (body weight, 220 ± 10 g), with animal license SCXK (Jin) 2015-0001, were supplied from Experiment Animal Center of Shanxi Medical University (Taiyuan, China). Animals were housed in a temperature-controlled room (25 ± 3 °C) with a 12 h light/dark cycle and relative humidity of 60–70%. The experiment was carried out in the Department of Biochemistry Laboratory at the Ocean University of China, and all disposals were in accordance with the guidelines of Shandong Province Experimental Animal Management Committee and National Institutes of Health Guide for the Care.

SD rats were anesthetized with pentobarbital sodium (100 mg/kg i.p.) and ovaries were removed to establish the classic postmenopausal osteoporosis rat model [38]. Two weeks after surgery, ovariectomy was verified by means of vaginal smear, then the animals were randomized and assigned into the model group, normal group, and two experimental groups of 6 rats each. Different doses of NAG (100 and 250 mg/kg body weight, in saline) were administered to animals in experimental groups by intragastric administration every day for 12 weeks. Animals in the model group and normal group were administered saline. After the last administration, rats fasted for 12 h. All venous blood was collected from the abdominal aorta under anesthesia, and the animals were then killed by cervical dislocation. Liver, spleen, and uterine horns of each animal were stripped intact and weighed to calculate organ indexes. Right femurs were cleaned from adjacent tissue and frozen at −20 °C for the bone mechanical property measurement. Left femurs were used for the measurement of bone mineral content, and left tibias were kept in formaldehyde for histological studies.

### 4.4. Biochemical Analyses 

Serum was isolated by centrifugation (2000× *g*) with 3K30 Laboratory Centrifuge (Sigma, Bremen, Germany). Supernatant was collected and stored at −20 °C for further analysis of ALP, calcium, and phosphorus levels following the manufacturer’s protocol. The left femurs were measured with a digital weighing balance after natural drying. The dried femurs were burned to ash at 800 °C for 5 h, and the ash weight was measured. Inorganic content rate was calculated by dry weight/ash weight × 100%. The calcium content of femur and tibia was determined with atomic absorption measurement. 

### 4.5. Measurement of Femoral Mechanical Strength 

The isolated right femurs were assessed with the three-point bending test using the Instron 1101 universal material testing machine (Canton, MA, USA). The midpoint of the right femur was placed on a holding device to create the three-point bending test. Specimens were placed on two supports that were separated by 20 mm. A displacement rate of 5 mm/min was selected for applying the loading vertically to mid-diaphysis on the anterior surface upward until a fracture occurred [39]. The central loading point was displaced, and the load was recorded until the specimen was broken. From the load-displacement curve, the maximum load (ultimate strength) and fracture deflection were obtained. 

### 4.6. Histological Examination of Tibias 

The left tibias were fixed in 4% neutral formaldehyde for 24 h, and decalcified in decalcifying liquid for 48 h. The tibia tissues were then embedded in paraffin wax and cut into 5 μm sections. The cross sections were stained with H & E, and longitudinal sections were stained with Masson trichrome stain for pathological studies. Trabecular bone areas were quantified by computer-aided software and the relative area of the normal group was adjusted to 100%. 

### 4.7. MC3T3-E1 Cell Culture and Osteoblast Proliferation 

The murine MC3T3-E1 osteoblast line was purchased from the Type Culture Collection of the Chinese Academy of Sciences (Shanghai, China). The cells were cultured in MEM-Alpha culture medium containing 10% FBS, 100 U/mL penicillin, and 100 μg/mL streptomycin, and incubated in an atmosphere of 5% CO_2_ at 37 °C. 

NAG was dissolved in MEM-Alpha medium (plus 10% FBS) and sterilized with 0.22 μm film filtration. Cell proliferation was evaluated using the MTT assay, which measured the metabolic reduction of MTT to formazan [40]. MC3T3-E1 osteoblasts (4 × 10^3^ cells/well in 96-well plates) were incubated with different concentrations of NAG (10, 50, 100, 250, and 500 μg/mL) for 24, 48, and 72 h. The control group and blank group were set up at the same time. Control groups contained medium only, and blank group contained medium without cells [41]. After NAG treatment, cell morphological changes were observed under microscope (T1-SM 100, Nikon Co., Tokyo, Japan). Then, MTT (5 mg/mL, 20 μL) was added to each well and incubated at 37 °C for 4 h to allow for the formation of formazan crystals. Next, dimethyl sulfoxide (DMSO; 150 μL) was added into each well to dissolve the dark blue crystals. Finally, absorbance at 492 nm was measured on a Multiskan Go 151 Microplate Scanning Spectrophotometer (Thermo Fisher Scientific, Inc., Waltham, MA, USA). Each assay was repeated three times and all experiments were performed in sextuplicate wells. Cell proliferation rate (PR, %) was calculated according to the formula below: (Absorbance of NAG treated groups − Absorbance of blank wells)/(Absorbance of control groups − Absorbance of blank wells) × 100%. 

### 4.8. Cellular ALP Activity Assay and Alizarin Red S Staining of Osteoblasts 

To investigate the effect of NAG on ALP activity in osteoblasts, MC3T3-E1 cells were seeded in 24-well culture plates (2 × 10^4^ cells/mL). Cells in experimental groups were incubated with 10% FBS medium supplemented with different concentrations of NAG (10, 100, and 500 μg/mL) for 3, 7, and 10 days. Cells in the control group were treated with MEM-Alpha medium (plus 10% FBS) only. At different time point, cells were washed three times with phosphate buffer solution (PBS) and lysed with 0.02% Triton X-100 by the freeze-thaw cycle. The total protein was extracted, and the concentration was detected using the Lowry method [42]. The ALP activity in cell lysate was measured using p-nitrophenyl phosphate as the substrate.

The mineralization of osteoblasts was assessed using Alizarin Red S staining [43]. MC3T3-E1 cells were seeded in 24-well plates at a density of 2 × 10^4^ cells/mL. After 24 h, cells were induced for differentiation with 25 μg/mL ascorbic acid (AA) and 5 mmol/L β-glycerophosphate (βGP) in 10% FBS medium, supplemented with various concentrations of NAG (10, 100, and 500 μg/mL). At 7 and 21 days, cells were washed with PBS twice and then fixed with 4.0% formaldehyde. The cells were stained with 40 mmol/L of Alizarin red S solution (pH 4.4) for 40 min at room temperature and rinsed with deionized water twice. The images of stained cells were captured using a phase contrast microscope with a digital camera (IM50, Leica, Germany). Cell mineralization was determined by measuring the absorbance of Alizarin Red S-stained cells at 570 nm [44]. 

### 4.9. Hydrogen Peroxide-Induced Oxidation Damage in Osteoblasts 

MC3T3-E1 cells (4 × 10^3^ cells/well in 96-well plates) were incubated with various dilutions of H_2_O_2_ (50, 100, 200, 300, and 400 μmol/L) for 2 and 6 h. MTT assay was adopted to select the appropriate damage concentration of H_2_O_2_. The cells were pretreated with NAG (10, 100 and 500 μg/mL) for 24 h, then the culture system was replaced with medium containing H_2_O_2_. Effects of NAG on H_2_O_2_-induced oxidative damage in osteoblasts were determined with calcein/PI fluorescence staining and MTT assay.

### 4.10. Statistical Analysis

All data are expressed as mean ± SD. Data analysis was carried out by the Student’s *t* test or the one-way analysis of variance (ANOVA) with SPSS version 18 (SPSS Inc., Chicago, IL, USA). * *p* < 0.05 was set as the significant level and ** *p* < 0.01 was considered very significant. 

## 5. Conclusions

Overall, the in vivo studies showed that NAG administration could reverse ovariectomized-induced bone loss, deteriorative microarchitecture, decreased bone strength, and impaired histologic structures of rats. In vitro studies revealed the proliferation stimulating and differentiation promotion effects of NAG on MC3T3-E1 cells. NAG suppressed oxidative stress and enhanced the antioxidant defense mechanism in osteoblasts. Our results suggest that the underlying mechanisms of NAG for PMOP treatment might be related to promoting osteoblasts, suppressing osteoclasts, and improving the content of body calcium. The specific mechanism still requires further illustration. The results implied that natural NAG obtained from snow-crab waste could be a promising candidate as new functional food ingredient to prevent PMOP.

## Figures and Tables

**Table 1:** Effects of *N*-acetyl-d-glucosamine (NAG) on the maximum load and fracture deflection.

Group	Maximum Load (N)		Fracture Deflection (mm)	
	8 Weeks	12 Weeks	8 Weeks	12 Weeks
Normal group	138.32 ± 4.44	163.97 ± 7.16	1.39 ± 0.05	1.59 ± 0.11
Model group	111.81 ± 6.84 ^##^	93.77 ± 9.4 ^##^	1.09 ± 0.02 ^##^	1.06 ± 0.03 ^##^
NAG 250 mg/kg	123.63 ± 5.32 ^##,^*	161.26 ± 8.33 **	1.22 ± 0.06 ^##,^**	1.42 ± 0.07 **
NAG 100 mg/kg	117.08 ± 12.36 ^##^	148.33 ± 6.38 ^#,^**	1.14 ± 0.10 ^##^	1.29 ± 0.11 ^##,^**

Data represent mean ± SD; *n* = 6; ^#^ *p* < 0.05, ^##^ *p* < 0.01: significant difference compared with normal group; * *p* < 0.05, ** *p* < 0.01: significant difference compared with model group.

**Table 2:** Effects of NAG on right femur dry weight, ash weight, and percentage of inorganic and content of mineral calcium at 12 weeks.

Group	Bone Mineral Content				
	Dry Weight (g)	Ash Weight (g)	Inorganic Content (%)	Femur Ca (mg/g)	Tibia Ca (mg/g)
Normal group	0.5271 ± 0.016	0.3427 ± 0.015	65.08%	196.96 ± 1.41	180.79 ± 7.84
Model group	0.4682 ± 0.017 ^##^	0.2612 ± 0.015 ^##^	56.78%	132.34 ± 1.35 ^##^	104.16 ± 7.6 ^##^
NAG 250 mg/kg	0.5226 ± 0.012 **	0.3445 ± 0.007 **	65.93%	202.5 ± 8.73 **	163.88 ± 9.96 **
NAG 100 mg/kg	0.4943 ± 0.014 ^#,^*	0.3022 ± 0.021 ^#,^*	61.14%	173.85 ± 13.56 ^#,^**	156.4 ± 8.36 ^##,^**

Data represent mean ± SD; *n* = 6; ^#^ *p* < 0.05, ^##^ *p* < 0.01: significant difference compared with normal group; * *p* < 0.05, ** *p* < 0.01: significant difference compared with model group.
